# Pediatric Tui Na for Feeding Intolerance in Premature Infants: Protocol for a Systematic Review and Meta-Analysis

**DOI:** 10.2196/46375

**Published:** 2023-10-16

**Authors:** Zirong Bai, Xiaoxiao Lyu, Yichuan Tang, Meng Wang

**Affiliations:** 1 Department of Rehabilitation College of Acupuncture and Moxibustion and Massage Health Preservation and Rehabilitation Nanjing University of Chinese Medicine Nanjing China; 2 Department of Medicine, Dentistry and Health Sciences University of Melbourne Melbourne Australia; 3 Department of Rehabilitation Second People's Hospital of Tibet Autonomous Region Tibet Autonomous Region China; 4 Faculty of Medicine and Health School of Health Sciences University of Sydney Sydney Australia

**Keywords:** feeding intolerance, pediatric tui na, premature infants, protocol, systematic review

## Abstract

**Background:**

Feeding intolerance (FI), frequently resulting from gastrointestinal immaturity, is prevalent among premature infants. Current practices are gradually prioritizing nonpharmacological treatments, such as massage or “Tui na,” considering the potential side effects of prolonged medication use. Pediatric Tui na, a specialized massage therapy based on traditional Chinese medicine, has been widely studied for treating FI in premature infants. However, to our knowledge, no systematic review specifically focusing on the effectiveness and safety of traditional Chinese medicine–based pediatric Tui na for FI in premature infants has been published yet.

**Objective:**

This study aims to develop a protocol for a systematic review and meta-analysis for evaluating the safety and efficacy of pediatric Tui na for premature infants with FI.

**Methods:**

We will perform a comprehensive search in the following databases: Springer, Cochrane Library, Embase, MEDLINE, Clarivate Analytics, Physiotherapy Evidence Database (PEDro), CINAHL, PubMed, Scopus, World Health Organization (WHO) International Clinical Trials Registry Platform, and Chinese biomedical databases (Wanfang database, the China National Knowledge Infrastructure, Chinese Scientific Journals Database, and Chinese Biomedical Literature Databases), limited to studies published in Chinese and English languages between January 2000 and January 2023. The search strategy will use MeSH (Medical Subject Headings) terms and database-specific keywords. A total of 2 independent reviewers will initially screen the studies based on titles and abstracts, followed by a full-text evaluation of the eligible studies. Studies will include any nonrandomized controlled trials, nonrandomized clinical studies, randomized controlled trials, and quasi-experimental studies wherein the treatment group involves premature infants with FI given pediatric Tui na. Primary outcomes will be necrotizing enterocolitis, gastric residual volume, emesis, and stool blood. Secondary outcomes will be abdominal distension weight gain, time to achieve full enteral feeding, any adverse effects associated with pediatric Tui na, and length of hospital stay. The Cochrane Collaboration Risk of Bias Tool will be used to assess the risk of bias and methodological quality. Funnel plots will be used for evaluating publication bias. Meta-analysis will be conducted using the Review Manager software (version 5.4; Cochrane Collaboration). Subgroup analyses will be considered according to treatment received, country or setting, sex, and birth weight of premature infants (if heterogeneity is high, *I*^2^≥50%).

**Results:**

This is a systematic review and meta-analysis protocol, so the results are not yet available. The protocol has been registered with PROSPERO (CRD42023390021). We are currently in the study selection phase. Results are expected to be completed by the end of 2023.

**Conclusions:**

Following this protocol, a comprehensive and rigorous literature synthesis will be developed to assess the impact of pediatric Tui na treatment on premature infants with FI, enabling the determination of its efficacy and safety.

**Trial Registration:**

PROSPERO CRD42023390021; https://tinyurl.com/bdf4kn23

**International Registered Report Identifier (IRRID):**

PRR1-10.2196/46375

## Introduction

Feeding intolerance (FI) is usually a consequence of gastrointestinal immaturity and commonly occurs in premature infants [1]. It typically presents as increased gastric residual volume (GRV), abdominal distention, vomiting, stool blood, and emesis [2], affecting 33.8%-53.45% of premature infants in China [3]. Furthermore, there is an association between high GRV and increased morbidity of necrotizing enterocolitis (NEC) [4,5]. These gastrointestinal immaturity–associated intestinal system problems can affect the nutritional intake and gut morbidity of premature infants, leading to several complications such as prolonged hospitalization and weight loss, and even increased mortality [6,7], thus placing a heavy burden on health care services.

There is no ideal or effective treatment available for FI as its pathobiology remains to be fully understood and identified [8-10]. Previous guidelines and studies have primarily focused on pharmacotherapies for managing FI [11]. However, long-term use of medications such as erythromycin, probiotics, metoclopramide, and cisapride has been reported to be associated with many adverse effects (AEs), including neurological disorders and cardiovascular dysfunction [12-16], leading to a greater expenditure of time and money and posing a significant burden on patients [10]. Consequently, current guidelines and practices increasingly focus on nonpharmacological treatments such as massage or Tui na [4,17]. Pediatric Tui na, based on the principles of the meridian theory [18] of traditional Chinese medicine (TCM) [19], is widely used as a complementary and alternative medicine in East Asia [20-23]. It treats a range of pediatric diseases by applying various manipulations on the body surface, including pressing, kneading, circling, nipping, and pushing [24,25]. Studies have indicated that Tui na and other similar touch therapies can regulate hormones such as cortisol and dopamine and accelerate blood and lymphatic circulation, thereby improving the function of the gastrointestinal system [3,21,22,26-29]. Many studies [24,25,29-32], especially those conducted in China, have explored pediatric Tui na as a treatment for FI in premature infants. However, the results of these studies are inconsistent. For instance, several investigations [24,25,30] have indicated that pediatric Tui na provides significant benefits to premature infants with FI, including improved weight gain; reduced time to achieve full enteral feeding; reduction in GRV, emesis, and abdominal distention; and a notable reduction in the average length of hospital stay. However, Liu and Zhang [31] did not find any significant difference between the control group and the pediatric Tui na group in terms of weight gain or the time required to return to birth weight (*P*>.05). Additionally, a meta-analysis [33] suggested that massage benefits premature infants with FI. However, this analysis was limited to studies published in Persian and English. Furthermore, it primarily evaluated the effects of massage on GRV and did not specifically address the efficacy and safety of pediatric Tui na regarding intestinal function and other FI-associated factors in premature infants. Therefore, despite the research mentioned above, to our knowledge, no studies have specifically addressed the safety and efficacy of pediatric Tui na in premature infants with FI, highlighting a gap in understanding its role as a nonpharmacological treatment for these infants. Moreover, no published systematic review, meta-analysis, or review protocol focusing on TCM-based pediatric Tui na for premature infants with FI is yet available.

Considering the high heterogeneity of previous studies and their varying outcomes, as well as the absence of any comprehensive evidence on this subject, it is crucial to assess the effectiveness and safety of pediatric Tui na for premature infants with FI. We intend to conduct a systematic review and meta-analysis to enhance the current evidence base on the effectiveness of pediatric Tui na for FI in premature infants. To ensure the most comprehensive data collection, minimize publication bias, and summarize the currently available evidence, we will conduct electronic searches for studies published both in English and Chinese.

## Methods

### Overview

The protocol for this review has been registered with the National Institute for Health Research (PROSPERO 2023 CRD42023390021) [34]. This protocol will be reported and conducted according to the PRISMA-P (Preferred Reporting Items for Systematic Review and Meta-Analysis Extension for Protocols) 2015 guidelines [35] and the PRISMA (Preferred Reporting Items for Systematic Review and Meta-Analysis) guidelines [36].

### Study Selection Criteria

The eligibility criteria of studies will be presented in the PICOS (Patient, Intervention, Comparison, Outcome, and Study) design framework [37,38].

### Types of Studies

The following types of studies will be included: (1) any nonrandomized clinical studies, nonrandomized controlled trials, randomized controlled trials, and quasi-experimental studies, wherein the treatment group involved premature infants with FI who received pediatric Tui na; (2) interventional studies, retrospective or prospective observational studies, or case series; and (3) studies that reported and included data associated with specific primary (NEC, GRV, emesis, abdominal distension, and stool) and secondary outcomes (weight gain, time to achieve full enteral feeding, length of hospital stay, and any AEs associated with pediatric Tui na). Single-case reports will not be included.

### Participants

On the basis of any definition of FI, we included studies in which premature infants received pediatric Tui na to prevent or treat FI. We will identify prematurely born infants as per the definition of the World Health Organization (WHO); this includes infants born within 259 days from the first date of the mother’s last menstrual period or before 37 full weeks of gestation [39].

### Intervention

We will include all trials evaluating pediatric Tui na intervention for FI.

### Comparison

We will include studies with different control interventions, such as placebo and other currently used interventions (eg, physiotherapy, acupuncture, gentle touch, and medication), and those without any control group.

### Outcomes

Emesis, NEC, GRV, and stool blood will be included as primary outcomes. Any AEs associated with pediatric Tui na, abdominal distension, time to achieve full enteral feeding, length of hospital stay, and weight gain will be included as secondary outcomes.

### Search Strategy

A comprehensive search will be performed in the following databases: Springer, Cochrane Library, Embase, MEDLINE, Clarivate Analytics, Physiotherapy Evidence Database (PEDro), CINAHL, PubMed, Scopus, WHO International Clinical Trials Registry Platform, and Chinese biomedical databases (Wanfang database, the China National Knowledge Infrastructure, Chinese Scientific Journals Database, and Chinese Biomedical Literature Databases), limited to studies published in Chinese and English languages between January 2000 and January 2023. The database searches will include only studies published in peer-reviewed journals and reporting results within their full text. Experts will be consulted for including additional studies. With inputs from the principal investigator of the study, an experienced librarian at Nanjing University of Chinese Medicine will design and carry out a comprehensive literature search. For searching publications on the safety and effect of pediatric Tui na on premature infants with FI, we will use controlled vocabulary and keywords. Table 1 shows the search strategy for the PubMed database.

**Table 1:** Search strategy for the PubMed database.

Number	Search terms
1	Feeding intolerance [MeSH^a^]
2	(Feeding intolerance OR feeding difficulties OR gastrointestinal motility OR gastroesophageal reflux OR gastric residuals, abdominal distension, OR emesis): ti, ab^b^
3	1 OR 2
4	Tui na therapy [MeSH]
5	(Tui na OR pediatric Tui na OR massage OR tactile-kinesthetic stimulation OR massaging OR massotherapy OR manipulation OR chiropractic therapy OR abdominal Tui na therapy OR spinal manipulation OR Tui na using a single thumb): ti, ab
6	4 OR 5
7	Randomised: ti, ab
8	Randomized: ti, ab
9	Randomly: ti, ab
10	Clinical trials [MeSH]
11	Trial: ti, ab
12	Randomized controlled trial [MeSH]: ti, ab
13	Randomized controlled trial [MeSH]: ti, ab
14	Controlled trial [MeSH]: ti, ab
15	7 OR 8 OR 9 OR 10 OR 11 OR 12 OR 13 OR 14
16	Premature infants
17	15 AND 16
18	3 AND 6 AND 17

^a^MeSH: Medical Subject Headings.

^b^ti, ab: terms in either title or abstract fields.

### Study Selection

**Figure 1:**
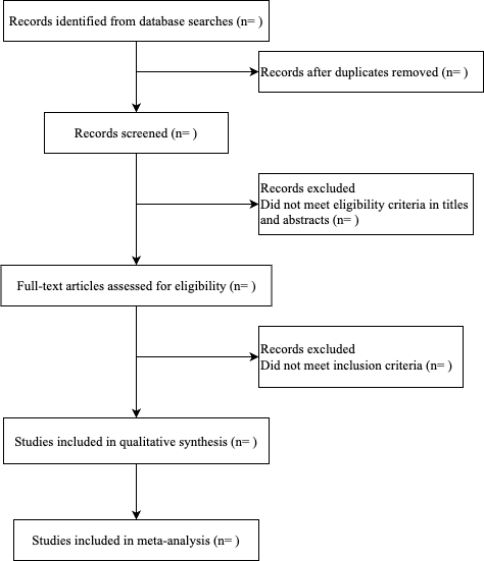
Flow diagram for study selection.

The retrieved literature will be imported into Endnote X9 (Clarivate Analytics); duplicate literature will be removed. A total of 2 authors will independently screen the titles and abstracts. Studies not meeting the inclusion criteria will be excluded. To seek full-text copies of studies or clarification of methods or results, study authors will be contacted through email if required. The latest or most comprehensive literature information will be extracted if multiple articles report the findings of the same study. Figure 1 shows the flow diagram for study selection.

### Methodological Quality and Risk of Bias Appraisal

A total of 2 researchers will evaluate the quality of the literature using the quality evaluation method of the Cochrane Collaboration System evaluator manual on the following 6 aspects [40]: concealment of the allocation scheme, generation of a random allocation scheme, complete data results, selective reporting results, blinding method, and other risks of bias.

### Data Extraction

A total of 2 independent reviewers will extract information using a predesigned form after identifying the studies to be included. The form will include data on the following aspects:

1. Identification information (first author, publication year, study design, country, and setting)
2. General information (study type, country, sample size, number of centers, and study design)
3. Participants (weight, sex, and age of premature infants)
4. Interventions (pediatric Tui na, type of pediatric Tui na, pediatric Tui na technique, pediatric Tui na position selection, and frequency and duration of sessions)
5. Comparator (if available, treatment details, including dosage, name, course, and frequency)
6. Outcomes (safety, efficacy data, and time points for each measurement)

If any information is missing or there is a need to seek further clarification, we will attempt to contact the corresponding authors.

### Assessment of Reporting Bias

We will use funnel plots to detect publication bias in the overall estimate and Egger and Begg tests to examine the funnel plot asymmetry. Symmetry and asymmetry in the funnel plot indicate low and high risks of reporting bias, respectively.

### Data Synthesis

Meta-analyses or descriptive analyses will be performed as per the measurement results, heterogeneity levels, and intervention measures. Conversely, we will report quantitative research results descriptively. Continuous variables will be analyzed according to mean differences and 95% CIs. Dichotomous variables will be assessed using risk ratios. Standardized mean differences and 95% CIs will be calculated if the outcome measure scales differ. Heterogeneity assessments will be performed within each pairwise comparison by the Cochran *Q* test and the *I*^2^ statistic. Negative *I*^2^ values will be treated as 0%, indicating no heterogeneity. *I*^2^<50% indicates low heterogeneity, while *I*^2^≥50% indicates high heterogeneity [41]. For sensitivity analysis, the results will be compared with those of other meta-analysis models, which would exclude trials with a high risk of bias and include fixed-effects models. We will perform a meta-analysis using a random-effects model if at least two included trials are found to be sufficiently homogeneous for the aspects of the comparator, outcome measurement, and study design. For performing the meta-analysis, the Cochrane Collaboration’s software Review Manager (version 5.4) will be used. Subgroup analyses will be considered according to the treatment received, country or setting, sex, and birth weight of premature infants (if heterogeneity is high, *I*^2^≥50%).

### Dissemination

This meta-analysis and systematic review will be submitted to a peer-reviewed journal. The findings are expected to shed some light on the efficacy and safety of pediatric Tui na for premature infants with FI.

## Results

This is a systematic review and meta-analysis protocol, so the results are not yet available. This protocol has been registered with PROSPERO (CRD42023390021) on February 8, 2023 [34]. We are currently in the study selection phase. Results are expected to be completed by the end of 2023.

## Discussion

This study aims to conduct a systematic review and meta-analysis of relevant research articles to comprehensively summarize the current evidence regarding the efficacy of pediatric Tui na for premature infants with FI. Pediatric Tui na, originated in China over 2300 years ago, was first recorded in *Wushierbingfang* (*Prescriptions for Fifty-two Diseases*) [42]. It is a commonly used, convenient, noninvasive, and cost-effective intervention therapy suitable for children [43]. Xie et al [44] reported that working on specific acupoints of the skin surface in pediatric Tui na can improve the gastrointestinal function of children. Xiao et al [24] and Chen [29] have reported that pediatric Tui na can accelerate gastrointestinal tract maturity, promote intestinal evacuation, and increase gastrointestinal motility in premature infants, thus alleviating FI. Although many clinical trials [24,25,30-32] have evaluated pediatric Tui na for treating FI in children, its efficacy and clinical evidence have not yet been established. The current clinical guidelines in China recommend massage therapy for treating FI in premature infants [17]. Despite being a TCM-based approach, pediatric Tui na is currently not included in the guidelines for treating FI in premature infants. This omission has prevented it from being widely and successfully adapted as a potential treatment for FI in premature infants. This study will be the first to evaluate the effect and safety of TCM-based pediatric Tui na for FI in premature infants by objectively synthesizing the currently available publications.

Some limitations should be considered in this systematic review. First, it will consider only studies published in Chinese and English, which could introduce potential language bias, resource constraints, difficulty locating non-English studies, and the possibility of bias in data extraction and analysis. Second, the specific focus of our research question might restrict the number of eligible studies for inclusion in the proposed review. Nonetheless, our objective is to extract valuable insights from the available evidence to offer preliminary guidance for current FI practices and future research. To stay updated with new evidence, we plan to conduct updates to the systematic review and meta-analysis every 3 years.

This protocol intends to aggregate current research, provide greater insights into the efficacy and safety of pediatric Tui na for FI, and offer a comprehensive assessment of the evidence for pediatric Tui na treatment of FI in premature infants for health care providers worldwide. Additionally, it will lay the groundwork for future research in pediatric Tui na and serve as a reference for the potential inclusion of pediatric Tui na in the treatment guidelines for premature infants with FI.

## Abbreviations

AE: adverse effect
FI: feeding intolerance
GRV: gastric residual volume
NEC: necrotizing enterocolitis
PEDro: Physiotherapy Evidence Database
PICOS: Patient, Intervention, Comparison, Outcome, and Study
PRISMA: Preferred Reporting Items for Systematic Review and Meta-Analysis
PRISMA-P: Preferred Reporting Items for Systematic Review and Meta-Analysis Extension for Protocols
TCM: traditional Chinese medicine
WHO: World Health Organization

## Data Availability

Data sharing is not applicable to this article as no data sets were generated or analyzed during this study.
